# Cognitive and social adaptation in autism spectrum disorder: A prospective cohort study

**DOI:** 10.1590/1516-3180.2023.0184.R1.16022024

**Published:** 2024-04-22

**Authors:** Márcia Regina Fumagalli Marteleto, Jacy Perissinoto

**Affiliations:** I PhD. Psychologist, Postgraduate Program in Human Communication Disorders, Department of Speech-Language Pathology and Audiology, Universidade Federal de São Paulo (UNIFESP), São Paulo (SP), Brazil.; II PhD. Speech Therapist, Associate Professor, Postgraduate Program in Human Communication Disorders, Department of Speech-Language Pathology and Audiology, Universidade Federal de São Paulo (UNIFESP), São Paulo (SP), Brazil.

**Keywords:** Cognitive Psychology, Social adjustment, Autism spectrum disorder, Social skills, Cognitive skills, Adaptive behavior, Autism

## Abstract

**BACKGROUND:**

During development, children face a number of demands and cognitive, behavioral, and social challenges necessary for growth. Cognitive skills make individuals competent and allow them to interact with their environment.

**OBJECTIVE:**

To identify the cognitive skills that promote better social insertion in children with autism spectrum disorder within 12 months.

**DESIGN AND SETTING:**

Prospective cohort study

**METHODS:**

In this study, 21 children aged 3–12 years were assessed, and their mothers were interviewed. Children were enrolled in regular or special autistic schools. Twelve months after the first assessment, the same children participated in the second assessment. In individual interviews, mothers provided data by answering the Vineland Adaptive Behavior Scale. Each child was assessed individually using the fourth edition of the Stanford Binet Intelligence Scale 4th Edition.

**RESULTS:**

In the first assessment, the Stanford Binet areas and total scores correlated with the communication domains, daily life abilities, socialization, and total score of the Vineland Scale. After 12 months, a correlation was observed between the Stanford Binet areas and the total and communication domains, daily life abilities, socialization, motor abilities, and total score on the Vineland Scale.

**CONCLUSION:**

Logic mathematics and memory promote better social insertion in children with autism spectrum disorder. General cognitive ability promotes communication.

## INTRODUCTION

During development, children face a number of demands and cognitive, behavioral, and social challenges necessary for growth. Child development involves these different aspects, and its purpose is to make children skilled in responding to their needs and the demands of their environment. The interrelation between these aspects makes the development a complex and intriguing process.^1,2^

From a cognitive standpoint, children develop flexibility in thought, the ability to come up with strategies for problem solving, and the capability to establish spatial, temporal, and causal relationships between objects.^3^ From a social standpoint, they develop social skills for communicating and performing activities of daily living that make them independent; they also develop behavior that helps them relate better to others.^4-6^

Intelligence is an important parameter in the structuring and dynamics of global child development.^7^ It is conceived as a general cognitive skill made up of mental abilities directed at social adaptation, such as language, logical-mathematical reasoning, visual-spatial reasoning, and short-term memory.^8^ Cognitive skills involve responses for problem solving.^9^ These responses are concretely expressed in the performance of daily activities and influence the performance of adaptive skills, such as communication, daily living skills, socialization, and motor skills, necessary for children to be socially included and experience personal autonomy.^2^

Qualitative failures in adaptation to social demands over time lead to diagnoses such as autism spectrum disorder (ASD).^10^ In these syndromes of global development abnormalities, adaptive disabilities go hand-in-hand with communication problems as well as interest and activity restrictions.^10^ The manifestations vary considerably with regard to the degree of severity and intellectual level of each child, which affects the performance of the daily activities necessary for autonomy.^11^ The difficulties in social adaptation found in children with ASD may be linked to failures in cognitive processes related to intelligence. Thus, it is supposed that correlations between cognitive and adaptive skills change throughout development, and specific cognitive skills promote greater social inclusion in children with these conditions.^12^

Cognitive profiles have revealed specific skills that interfere with the adaptive profiles of children with ASD.^13,14^

Executive functions enable mental manipulation of ideas, thinking before acting, facing new and unforeseen challenges, resisting temptations, and focusing. In this sense, executive functions generally comprise three components: working memory, cognitive flexibility, and inhibitory or self-control.^15^ Thus, the executive functions have been studied and highlighted as important in the development of the pragmatic dimension of language, because the integrated operation of these functions allows to maintain and update the conversation without losing relevant information arising from the manipulation of facts in working memory and inhibition of off-topic responses. This study found a positive correlation between executive functions and theory of mind, which are considered predictors of the severity of autism symptoms.^16^ Executive functions can be identified in the absence or scarcity of symbolic play in children with ASD as well as in the presence of restricted and repetitive patterns of interest and activity.^17^

Cognitive delay manifests as logical and intuitive reasoning deficits related to expressive language. These deficits interfere with knowledge of the world, interaction with people, and expression of their desires.^18^ Increased cognitive performance and Intelligence Quotient (IQ) influence expressive language skills and are not related to social communication skills or diagnosis of ASD.^18,19^ Cognitive skills such as low non-verbal quotient are also related to expressive language deficits. Studies have shown that nonverbal quotients predict patterns of increased simultaneous and longitudinal expressive language and sentence speech acquisition in children with ASD,^18^ although intellectual disabilities commonly co-occur with low expressive language in this condition.^20^

The literature investigated social skills, executive functions, and theory of mind in six (6) children diagnosed with ASD.^21^ They observed that the language development level of these children is important for the interaction and understanding of social processes, and that executive functions and social skills are directly influenced by the developmental level of verbal language.

The development of children’s memory is strongly linked to that of communication and oral language in children with ASD, and the range of memory in these children remains intact.^22,23^ However, recent studies have shown that children and adults with ASD have difficulties with short-term memory, especially serial order memory. This variation in memory function patterns cannot be explained by intelligence or language skills but is likely related to memory processing.^24^ Furthermore, recent research has shown that individuals with ASD demonstrate difficulty with verbal and non-verbal short-term memory, especially in serial order, but not with item retrieval.^25^ Visual memory is an important skill for children with ASD; however, it depends on the complexity of the stimulus, that is, whether they depend on verbal skills as well as on the effect of age and IQ.^22,26^

Some children with autism spectrum disorder have an impressive memory for memorizing, but the memorized information does not generalize to different contexts. When memory is stimulated by the environment, it becomes an important skill for social adaptation, as it facilitates communication and learning for activities of daily living, such as tying shoes, putting on clothes, buttoning shirt.^22^

The literature shows that deficits in divided attention and verbal fluency are related to social disabilities as well as impairments in working memory, which play a role in adaptive social difficulties.^27,28^

Another important aspect is that children with ASD show excellent performance in visual and spatial perceptual functions, such as detecting objects changing positions, known routes or paths, color discrimination, and simple visual tracking tasks. Although visuospatial perception may contribute to the high skills of these children,^29^ they present difficulties when asked, in this organizational process, to understand a sequence of images, both in sentence construction and telling a story as well as difficulty in linking sensory information into a unified perception.^30^

## OBJECTIVE

The aim of the present study was to identify the cognitive skills that promote greater social insertion in children with ASD on two separate evaluation occasions with a 12-month interval.

## METHOD

This prospective cohort study was approved by the Ethics Committee of the Universidade Federal de São Paulo (Brazil), with the authorization of the services involved (CEP no 0334/06, dated April 13, 2006). The parents/guardians of the participants signed informed consent forms.

### Participants

This study was conducted on two occasions. Twenty-six children aged between 3 and 12 years participated in the evaluation along with their mothers. All children had been previously diagnosed with ASD, confirmed by a specialized multidisciplinary team via interviews with parents, clinical evaluations, and based on the criteria of the Diagnostic and Statistical Manual of Mental Disorders Index (DSM-5).^10,31^ The children were involved in intervention programs at a specialized municipal school in São Paulo (Friends of Autism Association) and the Center for Speech and Hearing Investigation of Language in Global Development Disorders at the Teaching Clinic of the Sector of Human Communication Disorders, Department of Speech and Hearing Therapy, Universidade Federal de São Paulo. Twelve months after the first evaluation, 21 of the same mother/child pairs participated in the second evaluation, during which the children were aged between 4 and 13 years.

The inclusion criteria were as follows: boys and girls aged between 3 and 12 years (and their mothers) involved in regular public or private educational programs. The exclusion criteria were as follows: comorbidities such as hearing loss, encephalopathy, or tuberous sclerosis; non-adherence to the reassessment of the children 1 year after the beginning of the study; and abandonment of the educational program.

### Instruments

The Stanford Binet Intelligence Scale, 4th Edition^32^ was employed to measure children’s cognitive skills. This scale measures the general cognitive capacity and skills. It is made up of 15 subtests and furnishes an estimate of the general cognitive level and standard of cognitive skills in four areas: 1) Verbal Reasoning Subtests: Vocabulary, Comprehension, Absurdities, and Verbal Relations; 2) Visual-Abstract Reasoning Subtests: Pattern Analysis (Cubes), Copying, Matrices, Paper Folding, and Cutting; 3) Quantitative Reasoning Subtests: Quantitative, Numerical Series, Equation Building; 4) Short-Term Memory Subtests: Bead Memory, Memory for Sentences, Memory for Objects, and Memory for Digits.

All subtests were divided into levels beginning at 2 years of age. Each age group was determined using two items of approximately equal difficulty. The levels are composed of items arranged in increasing order of difficulty. The administration begins with the Vocabulary subtest, which uses chronological age and determines the mental age from the score the child achieves; the mental age then defines the starting point for the other tests. When a child makes a mistake in one of the items, the administration of the test should return to the items on the previous levels until two consecutive items are answered correctly. The test was administered until the child consecutively answered three of the four items or four items for two consecutive levels incorrectly. The scores achieved in each subtest were converted into standardized scores for age in the corresponding tables. The score was considered normal when the average was 50, and the standard deviation was 8. The composite standard age score (SAS) is considered normal when the average was 100, with a standard deviation of 16. The SAS of the Stanford Binet Intelligence Scale is equivalent to the intelligence quotient (QI) or general reasoning skills.^32^

The Vineland Adaptive Behavior Scale (VABS)^33^ was used to measure adaptive skills.

The VABS^33^ aims to assess the adaptive development of children and adolescents in their daily lives. Adaptive behavior on the scale is subdivided into four domains and sub-domains: 1) Communication (67 questions), subdivided into receptive, expressive, and written; 2) Daily Living Skills (92 questions), personal, domestic, and community skills; 3) Socialization (66 questions), interpersonal relationships, play and leisure time, and coping skills; and 4) Motor Skills (36 questions), gross and fine motor skills.

An optional index (36 questions) was designed to evaluate maladaptive behaviors such as obstinacy, impulsiveness, stubbornness, aggressiveness, anxiety, introversion, negativism, and mood swings.

The responses were scored in the following manner: 2 – usually, 1 – sometimes, 0 – never. N denotes no opportunity (does not apply), and DK is used when the respondent does not know the answer. The responses were recorded on the form. The raw scores obtained for each subdomain are converted into standardized scores for age in the corresponding tables. A normal score was defined as a mean score of 90–110 points. In the present study, the raw scores were not converted into standardized scores for age; we only considered correct answers. To obtain the profile of children with ASD from the total score and scores per category and subcategory, the administration of the scale commenced with item 1 on all sections. Higher scores on the VABS indicated greater social adaptation in children with ASD.

### Procedure

In the individual interviews, mothers provided data on adaptive skills in response to the VABS. An intellectual assessment was performed on each child individually by a psychologist with experience in the administration of intellectual tests and trained in the administration of the Stanford-Binet Intelligence Scale 4th edition.

### Statistical Analysis

Sample power was calculated to determine the precision and reliability of the samples. The test revealed a sample power of 83% for 21 children. Spearman’s correlation coefficient was calculated to correlate the domains and total of the VABS test, and the areas and total of the Stanford-Binet test; as this statistical method is non-parametric and can be applied in more general cases when non-normal distribution is presumed.^34^ Spearman’s correlation coefficient was calculated for the first evaluation, second evaluation and to determine differences between the two evaluation moments. Statistical significance was set at P < 0.05.

## RESULTS

The results demonstrated that children with ASD had specific cognitive skills that only correlated with specific adaptive domains in both evaluations. The tables below display the descriptive measures of the total Stanford-Binet and VABS for both evaluations, the values for the Spearman correlation coefficients (r) between the areas of the Stanford-Binet and VABS domains, and the p-values of the significance tests of these coefficients.

Table 1 displays the descriptive measures for the total Stanford-Binet and VABS at both evaluation times.

**Table 1:** Descriptive measures for the total of the Stanford-Binet and VABS in both evaluation times

Scales/instruments	1st evaluation	2nd evaluation	P value
	Mean (SD)	Mean (SD)	
**Stanford-Binet**	53.76 (67.15)	60.00 (73.50)	0.001
**VABS**	212.86 (84.47)	243.67 (83.49)	0.002

* Significant at P < 0.005; VABS = Vineland Adaptive Behavior Scale; SD = standard deviation.

Table 2 displays the values of the Spearman correlation coefficients for the areas and total of the Stanford-Binet with the domains and total VABS in the first evaluation.

**Table 2:** Spearman correlation coefficients between the areas and total of the Stanford-Binet and the domains and total of the VABS in the first evaluation

VABS	Stanford-Binet				
	Verbal Reasoning	Visual Abstract Reasoning	Quantitative Reasoning	Short Term Memory	Total
Communication	r = 0.90*	r = 0.75*	r = 0.78*	r = 0.85*	r = 0.87*
Daily Living Skills	r = 0.83*	r = 0.81*	r = 0.80*	r = 0.76*	r = 0.82*
Socialization	r = 0.75*	r = 0.70*	r = 0.79*	r = 0.73*	r = 0.78*
Motor Skills	r = 0.37	r = 0.47	r = 0.50	r = 0.39	**r = 0.42**
Behavior	r = 0.36	r = 0.36	r = 0.41	r = 0.34	**r = 0.37**
**Total**	r = 0.76*	r = 0.73*	r = 0.76*	r = 0.72*	r = 0.07*

* Significant at P < 0.001; VABS = Vineland Adaptive Behavior Scale.

All areas of the Stanford-Binet were correlated with Communication, Daily Living Skills, Socialization, and total VABS (P < 0.05), whereas the areas of the Stanford-Binet were not correlated with the Behavior Problems domain of the VABS (P > 0.05).

Table 3 displays the values of the Spearman correlation coefficients for the areas and total of the Stanford-Binet with the domains and total VABS in the second evaluation.

**Table 3:** Spearman correlation coefficients for the areas and total of the Stanford-Binet with the domains and total of the VABS in the second evaluation

VABS	Stanford-Binet				
	Verbal Reasoning	Visual Abstract Reasoning	Quantitative Reasoning	Short Term Memory	Total
Communication	r = 0.91*	r = 0.72*	r = 0.89*	r = 0.93*	r = 0.85*
Daily Living Skills	r = 0.78*	r = 0.68*	r = 0.79*	r = 0.82*	r = 0.73*
Socialization	r = 0.80*	r = 0.61*	r = 0.87*	r = 0.82*	r = 0.72*
Motor Skills	r = 0.70*	r = 0.71*	r = 0.66*	r = 0.71*	r = 0.70*
Behavior	r = -0.37	r = 0.23	r = -0.31	r = 0.36	**r = 0.24**
**Total**	r = 0.85*	r = 0.68*	r = 0.83*	r = 0.88*	r = 0.79*

* Significant at P < 0.001; VABS = Vineland Adaptive Behavior Scale.

All areas and the total Stanford- Binet had a direct correlation with Communication, Daily Living Skills, Socialization, Motor Skills domains, and total VABS (P < 0.05).

Table 4 shows the values of the Spearman correlation coefficients for the Stanford-Binet areas with the VABS domains for both evaluations.

**Table 4:** Spearman correlation coefficients for the areas of the Stanford-Binet with the domains of the VABS in both evaluations

VABS	Stanford-Binet				
	Verbal Reasoning	Visual Abstract Reasoning	Quantitative Reasoning	Short Term Memory	Total
Communication	r = 0.36	r = 0.16	r = 0.03	r = 0.36	r = 0.55*
Daily Living Skills	r = 0.14	r = 0.14	r = 0.46*	r = 0.06	**r = 0.03**
Socialization	r = -0.24	r = -0.16	r = 0.41	r = 0.07	**r = -0.08**
Motor Skills	r = 0.22	r = 0.15	r = 0.10	r = 0.32	**r = 0.11**
Behavior	r = 0.31	r = 0.21	r = 0.17	r = 0.34	**r = 0.05**
**Total**	**r = 0.18**	r = -0.44*	r = 0.45*	r = 0.54*	**r = 0.10**

* Significant at P≤ 0.05; VABS = Vineland Adaptive Behavior Scale.

The Visual-Abstract Reasoning area had an inverse correlation with total VABS (P = 0.04) over the course of 12 months. The Quantitative Reasoning area had a direct correlation with the Daily Living Skills domain (P = 0.03) and VABS total (P = 0.03) over the course of 12 months. The Short-Term Memory area had a direct correlation with the total VABS (P = 0.01) over the course of 12 months. The Stanford-Binet total score was directly correlated with the Communication domain (P = 0.01) over the course of 12 months. No correlations were observed between the areas and total of the Stanford-Binet with the Communication, Daily Living Skills, Socialization, Motor skills domains, and total VABS (P < 0.05) after 12 months.

## DISCUSSION

In the present study, cognitive skills were highly correlated with adaptive skills in children with ASD. The Short-Term Memory area correlated with all VABS domains, except for motor skills. It may be presumed that the information processed and stored in memory is not used for motor aspects. Children likely need to use frequent, repeated practice to memorize and store motor sequences.

Regarding short-term memory, children are able to organize the information received from the environment by encoding active memory into long-term memory. The improvement in this specific skill allowed information to be transferred from long-term memory to active memory and the processes of trial, elaboration, interpretation, and re-codification used by the children to become more socially adapted. The development of memory involved changes in the skills of recognition, recall, strategies, problem solving, metacognition, and information content.^35^

Over the course of 12 months, logical-mathematical cognitive skills and memory promoted better social inclusion in children with ASD. General cognitive skills promote improvement in communication. The performance of cognitive, behavioral, and adaptive skills changed significantly over the course of 12 months, and specific cognitive skills promoted social inclusion.

The Visual-Abstract Reasoning area was inversely correlated with the total VABS over the course of 12 months. In terms of the average performance on the test in this area, there was no significant increase in the mean values of either evaluation, although Visual-Abstract Reasoning is often mentioned in the literature as the specific cognitive skill with the greatest emphasis in children with ASD.^36,37^ This result indicates that greater use of visual-spatial skills denotes less social inclusion, regardless of the time of evaluation. Visual-Abstract Reasoning measures spatial skills; it is a prerequisite for environmental stimulation and is directly correlated with fluid intelligence. This is an innate biological skill that children perform to solve problems linked to visual-spatial-perceptive processes. Children do not require others to help or even teach them this skill. The Stanford-Binet tests are related to the visual-spatial process and are based on visual analysis and synthesis of geometric figures from the whole to the parts and vice versa. Children may have had difficulties with social adaptation because the tests were self-satisfying, interesting, and self-reinforcing. In other words, the visual stimuli contributed to social isolation.

The Quantitative Reasoning area correlated with Daily Living Skills and total VABS over the course of 12 months. Elementary logic operations involve the possibility of reconstituting the path taken by thought, that is, the reversibility of the reasoning carried out.^7^ The solution of a mathematical problem, or logical quantitative reasoning, is divided into the representation and solving of the problem. According to the literature, individuals may differ in their ability to correctly translate sentences that make up the problem.^12^ To represent the problem, a child needs to have linguistic knowledge for a verbal analysis of the mathematical problem solicited on the test and its quantitative relation with the response required. Knowledge of the world is necessary for a child to represent a quantity.

Children’s linguistic knowledge increases with age. To solve mathematical problems, a child requires strategic knowledge or planning. Daily living skills are related to habits of self-sufficiency that allow children to actively participate in the environment in which they live. Children in this study exhibited improvements in elementary logic operations. It may be hypothesized that the improvement in the reversibility of thought, linguistic knowledge, and perception of the world enabled these children to translate the mathematical propositions solicited on the test as well as integrate, plan, and execute the activities. Corroborating these findings, the fact that they were able to perform an elementary mathematical operation facilitated organizing their desk, placing the correct number of plates solicited, handling money, telling time, and considering the possibility of a car approaching when they crossed the street.^12^ No significant associations were found in the literature between attention, working memory, inhibitory control, thinking flexibility, and social skills; however, planning appeared to be associated with adaptive communication skills.^38^

The Short-Term Memory area was directly correlated with the total VABS over the course of 12 months. This finding corroborates a previous study,^22^ which reported that memory promoted adaptive behavior in children with ASD. With the retention of knowledge and recognition of the information to be used correctly, more organized thought allowed the use of memory in a more coherent and adequate fashion. In the present study, attention was found to be a key factor in improving short-term memory. Attention is the means by which humans actively process a limited amount of information through the senses, memory, and cognitive processes. There appears to be a limit to the amount of information on which we can concentrate our mental resources at any given time. When we diminish our attention to sensations, thoughts, and memories, we may focus on stimuli that interest us. Our results corroborate the previous studies^27,28^ that showed that attention probably opened paths for memory processes in children and adolescents with ASD and facilitated social adaptation.

The intellectual capability to use more general reasoning processes, relate to complex ideas, form abstract concepts, or perform mental operations when solving relatively new problems is directly correlated with improvements in the Communication domain. This indicates that the children with ASD in the present study used reasoning and mental operations to develop speech, diminish their difficulties in being with others, and pay attention to social cues. Such skills are necessary for the development of gestural and visual communication that precedes the manifestation of speech.^39^

## CONCLUSION

In summary, the present study provided evidence of specific skills that promote the social inclusion of children with ASD over time. The identification of these skills assists the clinical and educational operations of multidisciplinary teams regarding the cognitive failures that hinder the social adaptation process and are common to individuals with these conditions.
